# Primary atomization of shear-thinning liquid jets: a direct numerical simulation study

**DOI:** 10.1038/s41598-024-75553-4

**Published:** 2024-10-12

**Authors:** Marianne Abdelsayed, Elias Trautner, Jakob Berchtenbreiter, Markus Klein

**Affiliations:** University of the Bundeswehr Munich, Department of Aerospace Engineering, Institute of Applied Mathematics and Scientific Computing, Werner-Heisenberg-Weg 39, 85577 Neubiberg, Germany

**Keywords:** Engineering, Fluid dynamics

## Abstract

Using direct numerical simulation, the primary atomization of shear-thinning liquid jets into stagnant gas is investigated. Starting from a Newtonian configuration with material properties approximately corresponding to a Diesel injection, two hypothetical shear-thinning cases using the power-law and the Carreau-Yasuda models for the calculation of the apparent viscosity are investigated. A recently developed tracking algorithm is used to identify droplets newly formed from the core jet, as well as all other droplets in the computational domain, and a number of relevant droplet characteristics, such as droplet volume, surface area and center of mass, is recorded at each time step. This allows a comparison of droplet characteristics on the basis of probability density functions. It is observed that the shear-thinning behavior of the liquid phase, which is particularly relevant at the interface, influences the droplet volumes and shapes. While the mean viscosity differs significantly for the different cases, the first- and second-order velocity and volume fraction statistics remain nearly unchanged.

## Introduction

Atomization is relevant in many industrial applications, such as spray-painting, coating processes, fuel injection in combustion engines, as well as in natural processes, including waterfalls and ocean sprays^1^. Primary atomization, which refers to the initial disintegration of a liquid jet into droplets and ligaments, is a complex physical mechanism that remains poorly understood. The interaction of turbulence, surface tension and aerodynamic forces plays a crucial role in the size and spatial distribution of the droplets produced.

As computing power continues to improve significantly, direct numerical simulation (DNS) becomes viable for moderate Reynolds and Weber numbers. This allows studies targeted at enhancing the understanding of the atomization process. Extensive research has been devoted to studying the atomization of round liquid jets injected into stagnant gas. One of the first reported DNS of a Diesel spray at low Reynolds and Weber numbers was conducted by Leboissetier and Zaleski^2^. Ménard et al.^3^ used a Coupled Level Set and Volume of Fluid (CLSVOF) approach to study the dynamics of a Diesel jet at an increased Weber number. Herrmann^4^ investigated the droplet size distributions and their dependency on the grid resolution using the Refined Level Set Grid (RLSG) method. Grid-independent size distributions were observed for droplets with a diameter of several cells. Using highly resolved DNS, Shinjo and Umemura^5^ examined ligament and droplet formation for a Diesel jet. Zeng et al.^6^ studied the effect of evaporation on the primary breakup of a Diesel spray. Their findings indicate that evaporation leads to an acceleration and intensification of both ligament breakup and droplet generation. Hasslberger et al.^7^ performed a flow topology analysis using DNS of primary Diesel jet atomization. The authors observed that the flow topology behavior for the investigated configuration appears to be dominated by the Reynolds number. Crialesi-Esposito et al.^8^ evaluated the effect of isotropic and anisotropic turbulent inflow boundaries on the atomization process. Their study demonstrates that, although nozzle turbulence can accelerate the spray breakup, it cannot be used to control the atomization process if the turbulence parameters remain unchanged. An extensive investigation of primary droplet characteristics of a Diesel spray by Trautner et al.^9^ indicates that primary atomization plays a major role in the investigated configuration compared to secondary atomization. Recently, Srinivasan and Sinha^10^ examined the effect of the jet velocity profile on the degree of atomization. Other studies have focused on the injection of planar jets into stagnant air. Sander and Weigand^11^ extensively investigated the effect of the mean velocity profile at the nozzle exit on the primary atomization process. A detailed analysis of the processes leading to primary atomization, considering in particular aerodynamic effects, has been carried out by Desjardins and Pitsch^12^. Li and Soteriou^13^ performed DNS of round jets in gaseous crossflow at different density ratios. In addition, other thoroughly investigated configurations are swirl atomizers^14^ and two-phase mixing layers^15^.

Non-Newtonian fluids are involved in numerous atomization processes, particularly in spray painting and in agricultural sprays. However, the behavior of non-Newtonian jets is not fully understood yet. Ertl and Weigand^16^ investigated the effect of different inlet velocity profiles on the primary breakup of shear-thinning jets, demonstrating how the reduced viscosity in the jets enhances the atomization process. Zhu et al.^17^ identified a novel breakup mechanism for shear-thinning jets. A few studies focused on viscoelastic planar jets^18,19^.

The present study aims to analyze the effect of shear-thinning liquid behavior on the primary atomization process using DNS. Two shear-thinning cases, in which we employ the power-law and the Carreau-Yasuda models for the calculation of the shear-dependent viscosity, are compared with a Newtonian reference case. First- and second-order statistics obtained in the statistically steady-state are presented. A particular emphasis is placed on a detailed analysis of droplet characteristics. Using an algorithm by Trautner et al.^9^, primary droplets, defined as droplets newly formed directly from the liquid core jet, are identified at the instant of their formation. Simultaneously, droplet characteristics, such as droplet volume, surface area and center of mass, are recorded. This allows a direct comparison between the characteristics of primary droplets and all other droplets within the computational domain.

## Numerical methods and setup

### Governing equations and numerical framework

The simulations have been performed using the finite-volume based state-of-the-art two-phase solver “PArallel Robust Interface Simulator” (PARIS). Using the Volume-of-Fluid (VOF) method^20^, the interface between the different phases can be described using a marker function $\alpha$, which tracks the liquid volume fraction. The marker function $\alpha$ is

1 $$\begin{aligned} \alpha ={\left\{ \begin{array}{ll} 0 & \text {in gaseous phase,}\\ ]0,1[ & \text {in interface cells,}\\ 1 & \text {in liquid phase.} \end{array}\right. } \end{aligned}$$

First, the interface is reconstructed using a piecewise linear interface calculation (PLIC) approach within the framework of the geometric VOF method. Then, the marker function is advected using the transport equation

2 $$\begin{aligned} \dfrac{\partial {\alpha }}{\partial {t}}+u_i\dfrac{\partial {\alpha }}{\partial {x_i}}=0, \end{aligned}$$

where $u_i$ denotes the $i^{th}$ component of the velocity vector. Material properties, namely the density $\rho$ and the dynamic viscosity $\mu$, are linearly interpolated using the marker function $\alpha$, i.e.,

3 $$\begin{aligned} \rho= & \alpha \rho _l+(1-\alpha )\rho _g, \end{aligned}$$

4 $$\begin{aligned} \mu= & \alpha \mu _l+(1-\alpha )\mu _g. \end{aligned}$$

Here, the indices *l* and *g* refer to the liquid and gas phase, respectively.

The incompressible Navier-Stokes equations for mass and momentum conservation based on the one-fluid formulation^21^, which uses a a single set of equations, is used to describe the fluid motion in both phases:

5 $$\begin{aligned} & \dfrac{\partial {u_i}}{\partial {x_i}}=0, \end{aligned}$$

6 $$\begin{aligned} & \rho \left( \dfrac{\partial {u_i}}{\partial {t}}+\dfrac{\partial {u_{i}u_{j}}}{\partial {x_j}}\right) =-\dfrac{\partial {p}}{\partial {x_i}}+ \dfrac{\partial }{\partial {x_j}}\left[ \mu \left( \dfrac{\partial {u_i}}{\partial {x_j}}+\dfrac{\partial {u_j}}{\partial {x_i}}\right) \right] +\sigma n_{i}\kappa \delta _S. \end{aligned}$$

Here, *p* is the pressure, $\sigma$ is the surface tension coefficient, $n_i=(\partial \alpha /\partial x_i)/|\nabla \alpha |$ is the liquid-phase oriented unit vector normal to the interface, $\kappa =\partial n_i/\partial x_i$ is the local interface curvature, $\delta _S=|\nabla \alpha |$ is the interface indicator function and the subscripts $i,j=1,2,3$ represent the Cartesian indices. The last term on the right hand side of Eq. 6 is the surface tension term, which is modeled using the Continuous Surface Force (CSF) approach^22^. The CSF approach incorporates surface tension by adding a corresponding force term to the Navier-Stokes equations. In this context, the surface tension is interpreted as a continuous effect across the interface rather than a boundary value condition. The local interface curvature is computed using a state-of-the-art approach by Popinet^23^.

Spatial discretization is performed on a staggered grid of Marker and Cell (MAC) type^24^, in which the velocity variables are stored on cell faces while all other variables are stored in the cell center. The Quadratic Upstream Interpolation for Convective Kinematics (QUICK) scheme^25^ has been employed for momentum advection, while the diffusive term of the momentum equation has been discretized using second-order central differencing. A red-black Gauss-Seidel solver with overrelaxation is used to solve the Poisson equation^26^. Time integration is performed using a second-order predictor-corrector method. The system of equations is solved in the framework of the projection method^27^. Firstly, a temporary velocity field is calculated by neglecting the pressure field. In the second step, the velocity field is corrected by adding the pressure field, resulting in a divergence-free velocity field.

### Case definition

The investigated jets are characterized by a Reynolds and Weber number of $Re=\rho _{l}U_{0}D/\mu _{l,0}=8,000$ and $We=\rho _{l}U_{0}^{2}D/\sigma =5,000$, respectively. In this context, $U_{0}$ is the injection velocity, *D* is the nozzle diameter and $\mu _{l,0}$ is the Newtonian dynamic viscosity. This moderate choice of Reynolds and Weber numbers results in the formation of a sufficient number of droplets, which is required for a meaningful statistical analysis of the droplet characteristics, while simultaneously maintaining the resolution requirements for DNS^9^. The density and dynamic viscosity ratios are $\rho _l/\rho _g=\mu _{l,0}/\mu _g=40$, which corresponds to the configuration shown in the work of Trautner et al.^9^. Following Stanley et al.^28^, the axial velocity profile of the round jets at the inflow is given by

7 $$\begin{aligned} U =\dfrac{U_0}{2} - \dfrac{U_0}{2}\cdot \text {tanh}\left( \dfrac{\sqrt{(y-y_c)^2+(z-z_c)^2}-\frac{D}{2}}{2\theta }\right) , \end{aligned}$$

where $\theta =D/20$ is the momentum thickness and $(y_c,z_c)$ denotes the center of the nozzle. The velocity profile at the inflow is superimposed by turbulent fluctuations with a turbulent intensity of $u'/U_0=7.5\%$ and an integral turbulent length scale $L_t=D/8$ using the digital filter method by Klein et al.^29^. At the outflow, zero gradient boundary conditions are used for both *p* and $\alpha$, and negative velocities are clipped to zero to avoid backflow. At the lateral boundaries, homogeneous Neumann conditions are considered. Moreover, the velocity field is filtered in a narrow band at these boundaries for stability reasons. The computational domain is a box with the dimensions $16D\times 6D\times 6D$. The mesh is cubic and equidistant, with a grid resolution of $\Delta =D/128$ and approximately $1.2\times 10^9$ cells. Based on Kolmogorov’s universal equilibrium theory, the estimation of the smallest dissipative length scale $\eta _K$ can be expressed as a function of $L_t$ and the turbulent Reynolds number $Re_t=u'L_t/\nu _{l,0}$^30^, in which $\nu _{l,0}$ denotes the Newtonian kinematic viscosity of the liquid phase:

8 $$\begin{aligned} \eta _K\approx L_t Re_t^{-3/4}. \end{aligned}$$

This yields $\eta _K/\Delta \approx 0.59$. Hence, the grid resolution is in the same order of magnitude as the Kolmogorov scale. A separate evaluation has revealed that this also holds true when considering the lower viscosity for the non-Newtonian sprays, and in particular the Carreau-Yasuda case (see Fig. 4). The chosen grid resolution reflects the state of the art^4,5,15,31^ of DNS atomization studies and is higher than the one used for comparable shear-thinning DNS atomization studies^16,17^. A higher grid resolution for studies in the statistically steady-state is currently infeasible due to the high computational cost.

To model the shear-thinning flow behavior of the liquid phase, the power-law and the Carreau-Yasuda models are used to account for the change in viscosity. The power-law model is widely used for its simplicity as it only has two parameters. Here, the apparent viscosity is expressed as^32^

9 $$\begin{aligned} \mu _{ap,PL}=K \dot{\gamma } ^{n-1}, \end{aligned}$$

where *K* is the consistency index, $\dot{\gamma }=\sqrt{\left( e_{ij}e_{ij}/2\right) }$ is the shear rate, $e_{ij}=\left( \partial u_i/\partial x_j+\partial u_j/\partial x_i\right)$ is the rate of strain tensor and *n* is the power-law index. For $n=1$ the flow is Newtonian and $\mu _{ap}$ corresponds to *K*. For $n<1$ ($n>1$) the flow is shear-thinning (shear-thickening). Compared to the power-law model, the Carreau-Yasuda model gives more flexibility to fit viscosity data, as it has more parameters. The apparent viscosity can be expressed as^32^

10 $$\begin{aligned} \mu _{ap,CY}=\mu _\infty +\left( \mu _0-\mu _\infty \right) \left[ 1+\left( \lambda \dot{\gamma }\right) ^a\right] ^{(n-1)/a}. \end{aligned}$$

Here, $\mu _0$ is the zero-shear viscosity, $\mu _\infty$ is the infinite-shear viscosity, $\lambda$ is the characteristic time and *a* describes the onset of the power-law region. Since the apparent viscosity is bounded by $\mu _0$ at low shear rates and by $\mu _\infty$ at high shear rates, the model gives more realistic values of the apparent viscosity at low and high shear rates compared to the power-law model^33^.

Table 1 provides the viscosity model parameters used in this study. As in other numerical studies aiming to investigate the effect of non-Newtonian material behavior^34,35^, the two shear-thinning fluids chosen in this study are hypothetical fluids. The model parameters have been selected in a manner that avoids an overly strong equivalence in the shear-thinning behavior of the power-law and the Carreau-Yasuda cases. For example, if the quasi Newtonian behavior of the Carreau-Yasuda case at very low shear rates would be extended to larger shear rates, the power-law and Carreau-Yasuda cases would exhibit a similar shear-thinning effect at higher shear rates, which are more likely to occur in the case of atomization than very low shear rates. In addition, the Newtonian and the Carreau-Yasuda cases would behave nearly identical for a large range of shear rates. The power-law index is chosen based on a previous study of turbulent bubble-laden channel flow of power-law fluids by Bräuer et al.^36^. In this study, a nearly identical numerical method is used to describe immiscible two-phase flow. It is reported that $n=0.7$ is sufficient to capture the shear-thinning characteristics of the flow. The infinite-shear viscosity is set to a very low value, as Carreau et al.^37^ noted that for most engineering applications $\mu _\infty$ is either set to a value of the order of the solvent or is minimal and can be considered negligible. The investigated sprays do not represent a specific real-world application. Nevertheless, the Carreau-Yasuda model parameters qualitatively resemble the behavior of the real fluids investigated experimentally by Ertl et al.^16,38^.

**Table 1:** Overview of the model parameters determining the non-Newtonian material behavior.

Case	$K [\mathrm {Pa\cdot s^n}]$	$n [-]$	$\mu _0 [\mathrm {Pa\cdot s}]$	$\mu _\infty [\mathrm {Pa\cdot s}]$	$a [-]$	$\lambda [\textrm{s}]$
Power-law	$1.156\cdot 10^{-4}$	$0.7$	−	−	−	−
Carreau-Yasuda	−	$0.7$	$1.156\cdot 10^{-4}$	$1.0\cdot 10^{-7}$	$1$	$20$

**Fig. 1:**
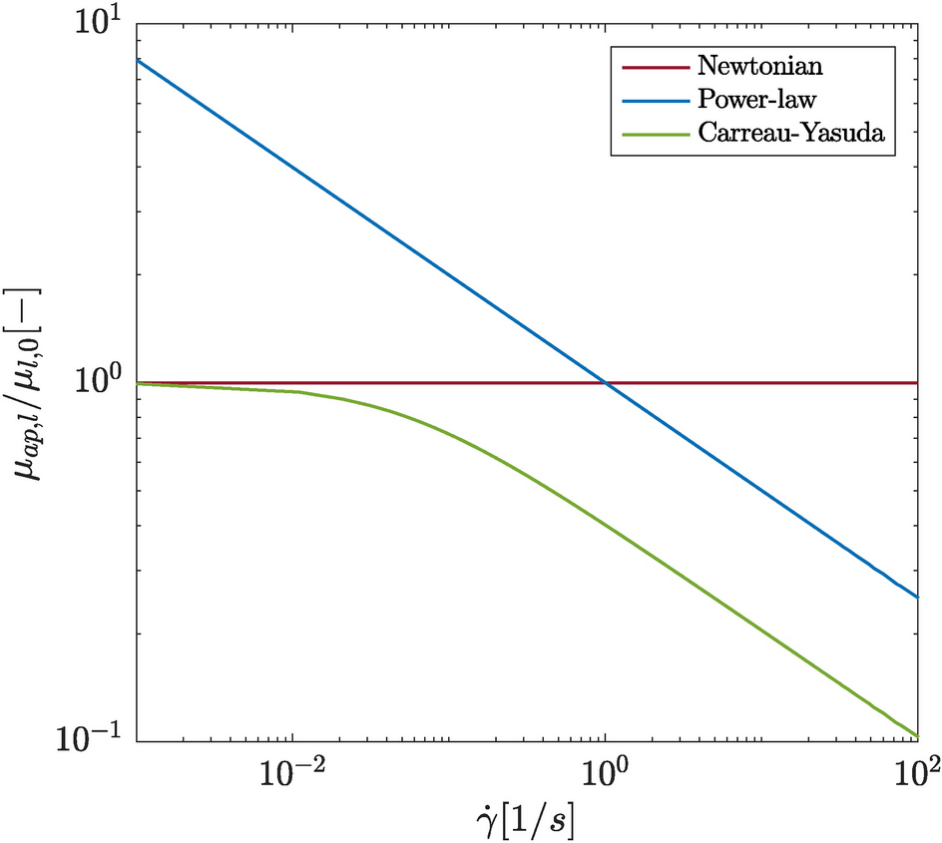
Apparent dynamic viscosity of the liquid phase $\mu _{ap,l}$ for all investigated cases normalized by the Newtonian dynamic viscosity $\mu _{l,0}$ as a function of the shear rate.

Figure 1 shows the dependency of the normalized apparent viscosity $\mu _{ap}$ of the liquid phase on the shear rate for all three investigated cases. Only the relevant range of shear rates is displayed. The apparent viscosity can be interpreted as the effective local viscosity. In Newtonian flows, the apparent viscosity remains constant, while in shear-thinning flows, it decreases with increasing shear rate. In two-phase flows, where the liquid phase exhibits a shear-thinning behavior, the apparent viscosity represents a crucial parameter which can exhibit a significant impact on the breakup characteristics. It can be observed that the Carreau-Yasuda case displays a stronger shear-thinning behavior than the power-law case. In this study, $\mu _{ap,PL}$ is not limited by a minimum or a maximum value.

### Identification of primary droplets

A recently proposed algorithm by Trautner et al.^9^, which is briefly explained below, is used in this study to identify primary droplets during the simulation. This process is carried out in the statistically steady-state for approximately one flow-through time. The following procedure is repeated at every time step and does not influence the outcome of the simulation.

At the beginning of a new time step, all cells either containing the $\alpha =0.5$ iso-contour or enclosed by it are identified. These cells are stored in a separate VOF sub-field, denoted as $\alpha _{tag}$, which is determined from the original VOF field $\alpha$. All other cells belong to smeared-out interfacial structures and are stored in a separate field $\alpha _{untag}$.

By using the parallel tagging algorithm of Herrmann^39^, which has been implemented and validated in PARIS by Ling et al.^40^, cells belonging to $\alpha _{tag}$ are either assigned to the liquid core jet or to the droplets surrounding it. This allows to further distinguish $\alpha _{tag}$ into $\alpha _{core}$ and $\alpha _{drop}$. A fourth sub-field $\alpha _{mix}$ accounts for the mixing of liquid volume stemming from $\alpha _{core}$ and $\alpha _{drop}$ in cells during the split-direction VOF advection performed in every time step. It should be noted that $\alpha _{mix}$ is set to zero at the beginning of each time step. The VOF field can thus be expressed as

11 $$\begin{aligned} \alpha (i,j,k)=\alpha _{core}(i,j,k)+\alpha _{drop}(i,j,k) +\alpha _{mix}(i,j,k)+\alpha _{untag}(i,j,k). \end{aligned}$$

In cells, where $\alpha (i,j,k)>0$, only one field on the right-hand side of Eq. 11 is positive and equal to $\alpha (i,j,k)$. In each time step, the separate VOF sub-fields are advected analogous to the full VOF field. The identification of newly formed primary droplets is based on the evolution of $\alpha_{core}$, whereas the other sub-fields are required for an advection consistent to the one of the full VOF field.

At the end of the time step, the algorithm by Herrmann^39^ is used again to detect liquid structures that have separated from the liquid core jet ($\alpha_{core}$) within that time step. These structures represent the newly created primary droplets. For a more detailed explanation of the algorithm, the reader is referred to Trautner et al.^9^.

## Results and discussion

In this section, the effect of the shear-thinning viscosity behavior of the liquid phase on the primary atomization process is discussed. The behavior of the sprays is investigated on the basis of first- and second-order statistics of the flow. This is followed by a detailed discussion on primary droplet characteristics, such as the spatial distribution of newly formed droplets, as well as droplet sizes and shapes.

**Fig. 2:**
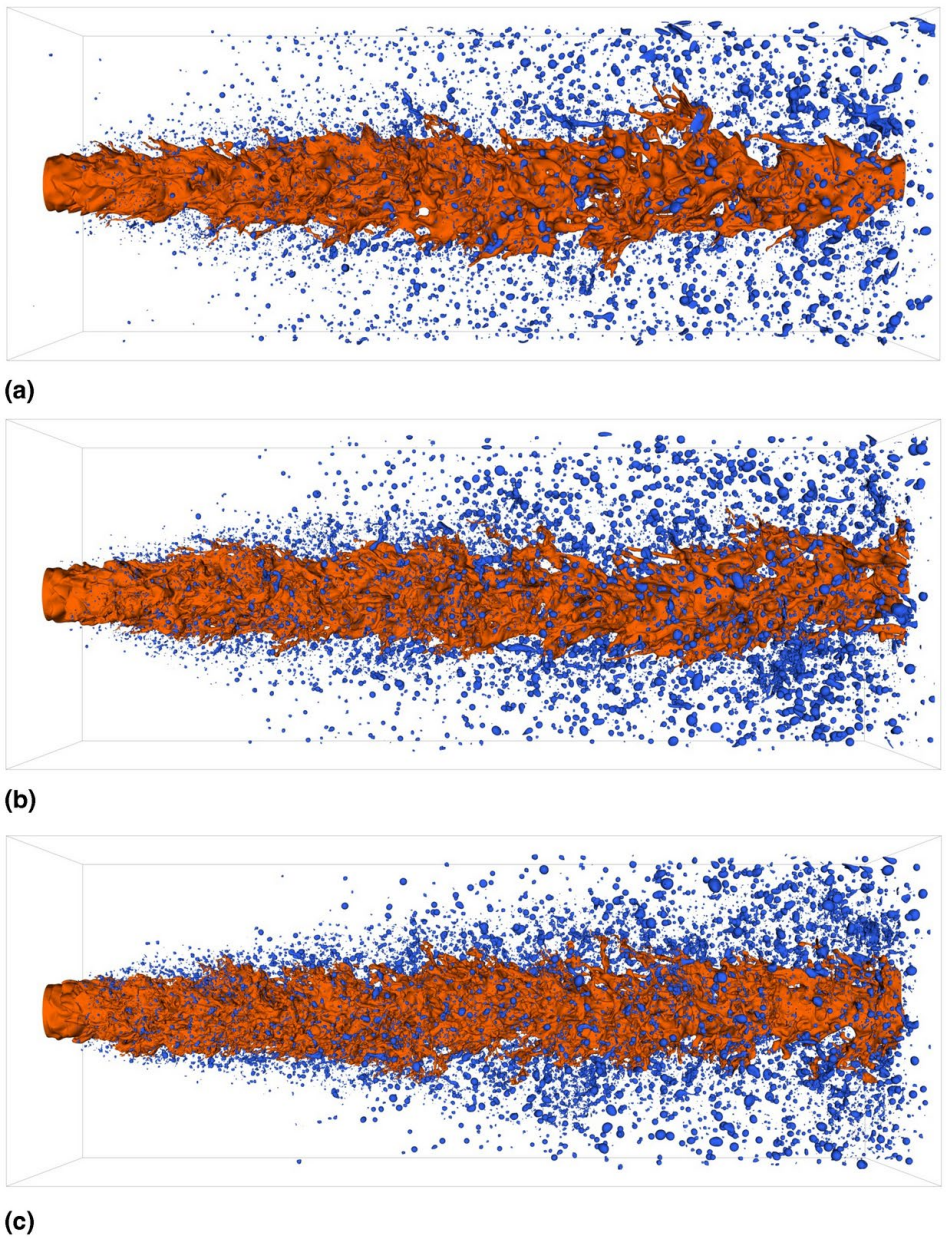
Iso-contours of the liquid phase volume fraction corresponding to $\alpha =0.5$. The orange and blue colors represent the jet core and all detected droplets in the computational domain, respectively. (**a**) Newtonian; (**b**) Power-law; (**c**) Carreau-Yasuda. The iso-contour plot of the Newtonian case has been reprinted from the International Journal of Multiphase Flow 160, 104360, E. Trautner, J. Hasslberger, S. Ketterl, M. Klein, “Primary atomization of liquid jets: Identification and investigation of droplets at the instant of their formation using direct numerical simulation”, Page 5, 2023, with permission from Elsevier^9^.

Figure 2 shows the $\alpha =0.5$ iso-contour of both the jet core (orange) and the complete droplet collective within the domain (blue) for the investigated cases. The Newtonian configuration shows first deformations of the jet core surface, as well as few small droplets near the nozzle. Further downstream, the spray is dominated by rather big droplets. The shear-thinning cases display a higher density of droplets compared to the Newtonian spray. This is particularly the case for the Carreau-Yasuda spray. Compared to the Newtonian case, the radial spreading in the immediate near-nozzle region is smaller for the non-Newtonian sprays. Similar to the Newtonian spray, larger droplets and ligaments dominate further downstream.

**Fig. 3:**
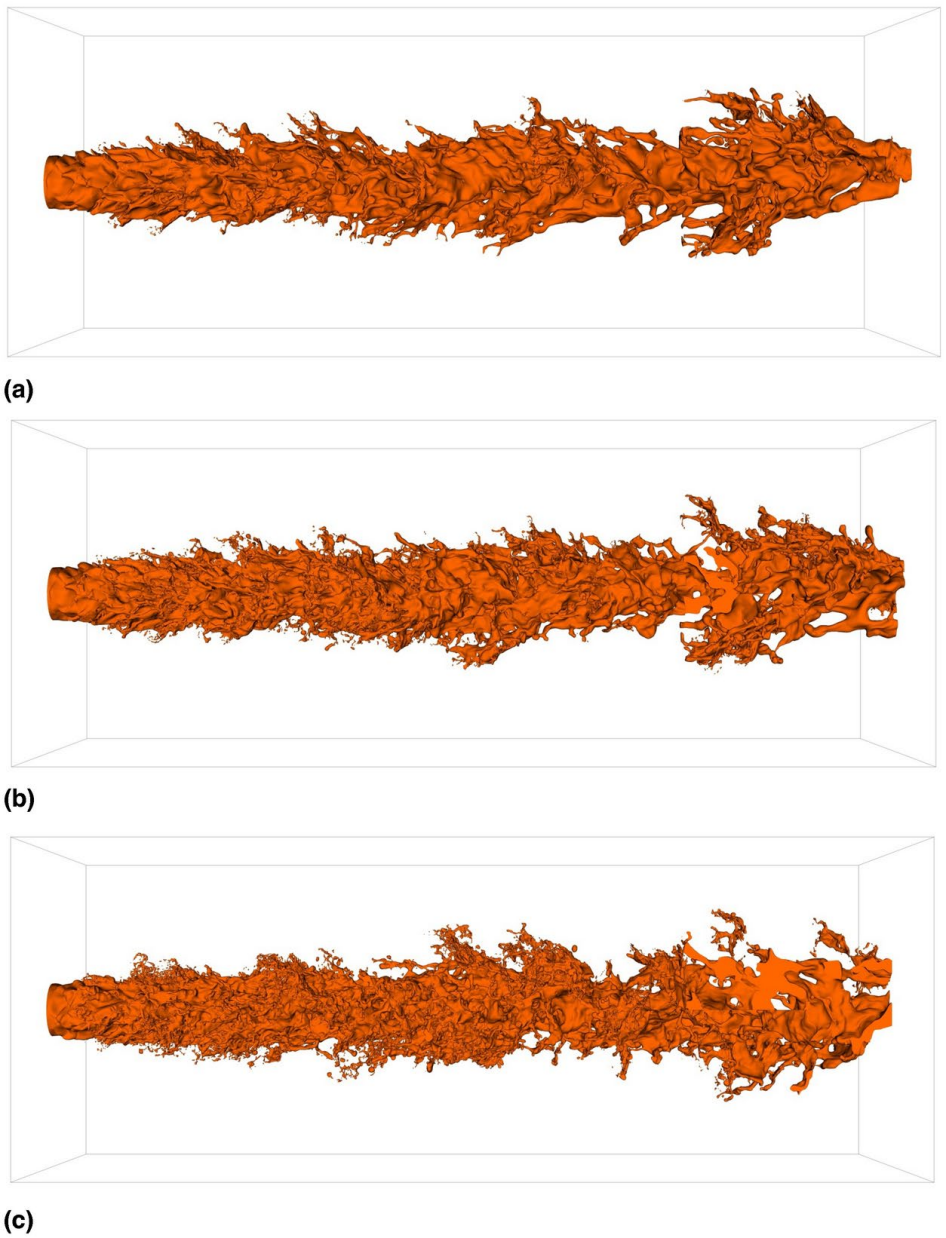
Iso-contours of the liquid phase volume fraction corresponding to $\alpha =0.5$. Only the jet core is displayed after removing all droplets in the computational domain. (**a**) Newtonian; (**b**) Power-law; (**c**) Carreau-Yasuda.

To evaluate the effect of the shear-thinning behavior of the liquid phase on the core jet, Fig. 3 illustrates the core jets for all investigated cases as an iso-contour of the liquid phase volume fraction corresponding to $\alpha =0.5$. Compared to Fig. 2, the droplets have been removed for increased clarity. Figure 3 demonstrates that, with increasing shear-thinning character of the liquid phase, the core jets exhibit an increasingly irregular and distorted surface, characterized by small surface structures. For a quantitative analysis of the jet core, the temporal average of the surface area as well as the volume of the core are given in Table 2. The surface area of the jet core increases with the shear-thinning character of the liquid phase, indicating a stronger breakup and stronger deformations of the jet core. This is consistent with the iso-contour plots presented in Fig. 3. Furthermore, as the shear-thinning character of the flow increases, the volume of the jet core decreases as the breakup intensifies and a greater number of droplets are formed.

**Table 2:** Temporal average of the normalized surface area and volume of the jet core for all investigated cases.

Case	$\langle A\rangle _t/D^2$	$\langle V\rangle _t/D^3$
Newtonian	$261.79$	$12.24$
Power-law	$306.68$	$12.11$
Carreau-Yasuda	$357.30$	$12.07$

**Fig. 4:**
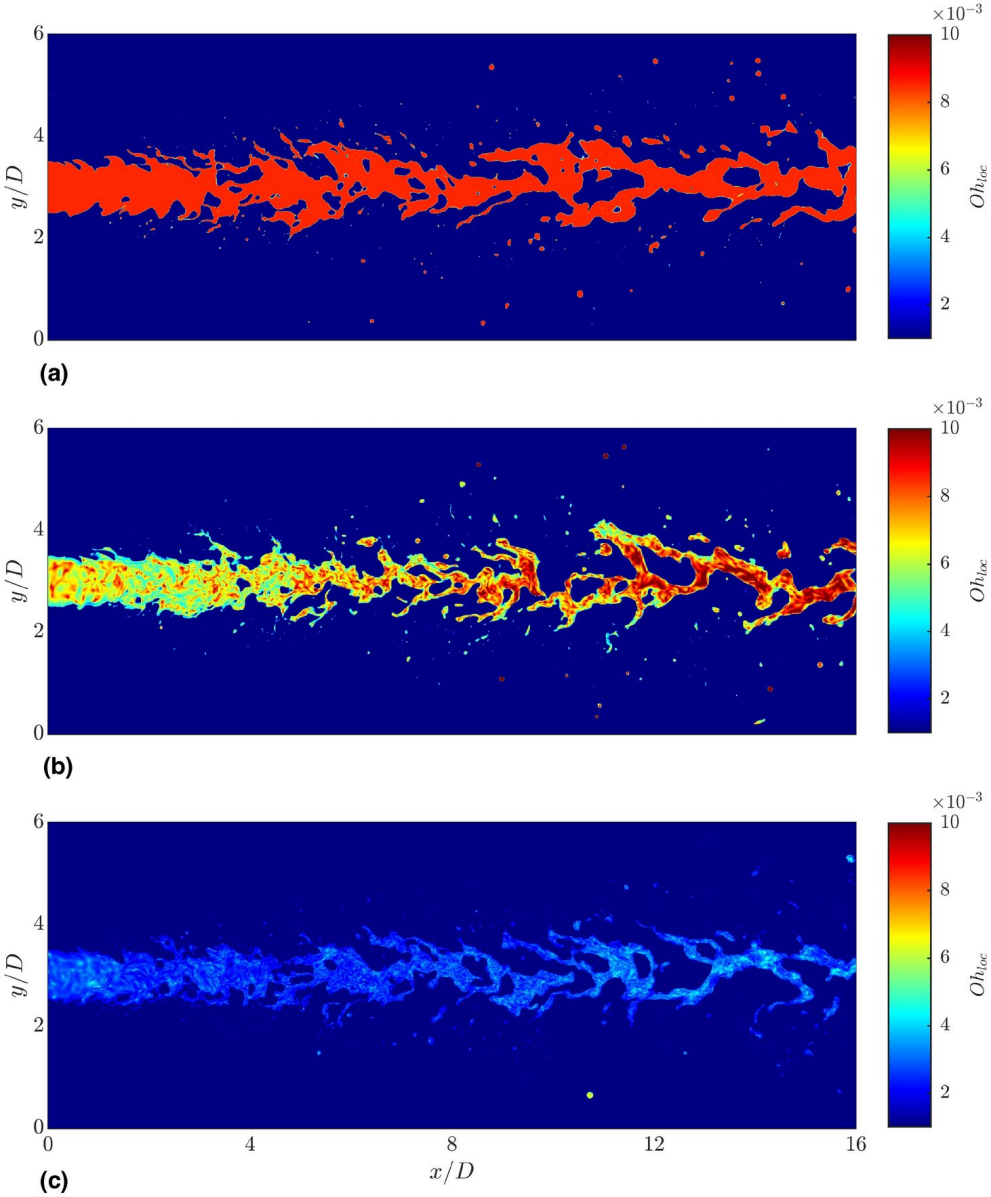
Instantaneous slice of the local Ohnesorge number $Oh_{loc}=\mu /(\sqrt{\rho _l D\sigma })$ in the midplane of the computational domain. Higher (lower) $Oh_{loc}$ indicate higher (lower) viscosities and thus lower (higher) shear rates in the shear-thinning cases. (**a**) Newtonian; (**b**) Power-law; (**c**) Carreau-Yasuda.

To qualitatively evaluate the shear-thinning flow behavior on the local apparent viscosity of the jet, Fig. 4 presents a centered vertical slice of the instantaneous local Ohnesorge number $Oh_{loc}=\mu /\sqrt{\rho _l D \sigma }$ for all investigated cases. The local Ohnesorge number, which represents the ratio of viscous to surface tension forces, can be interpreted as a non-dimensional local viscosity and thereby provides insight into the impact of shear-thinning behavior. In the Newtonian case, $Oh_{loc}$ is constant in both the liquid and gas bulk phases, as the viscosity only depends on the local value of the VOF field. However, when dealing with shear-thinning fluids, $Oh_{loc}$ locally varies with the shear rate in the liquid phase. Compared to the Newtonian case, which exhibits a constant value of $Oh_{loc}=8.5\cdot 10^{-3}$ in the liquid phase, the power-law case mostly exhibits lower $Oh_{loc}$ in the liquid jet, particularly in the first half of the domain and near the interface. This indicates high shear rates. In the second half of the domain, where turbulence has weakened, $Oh_{loc}$ partly exhibits higher values than in the Newtonian case in the jet center. This suggests low shear rates $\dot{\gamma }<1$, for which the power-law model predicts a viscosity higher than $\mu _{l,0}$. In contrast to the power-law case, the Carreau-Yasuda spray exhibits significantly lower values of $Oh_{loc}$ throughout the jet, as the liquid phase exhibits a stronger shear-thinning behavior than in the power-law case.

### First- and second-order flow statistics

**Fig. 5:**
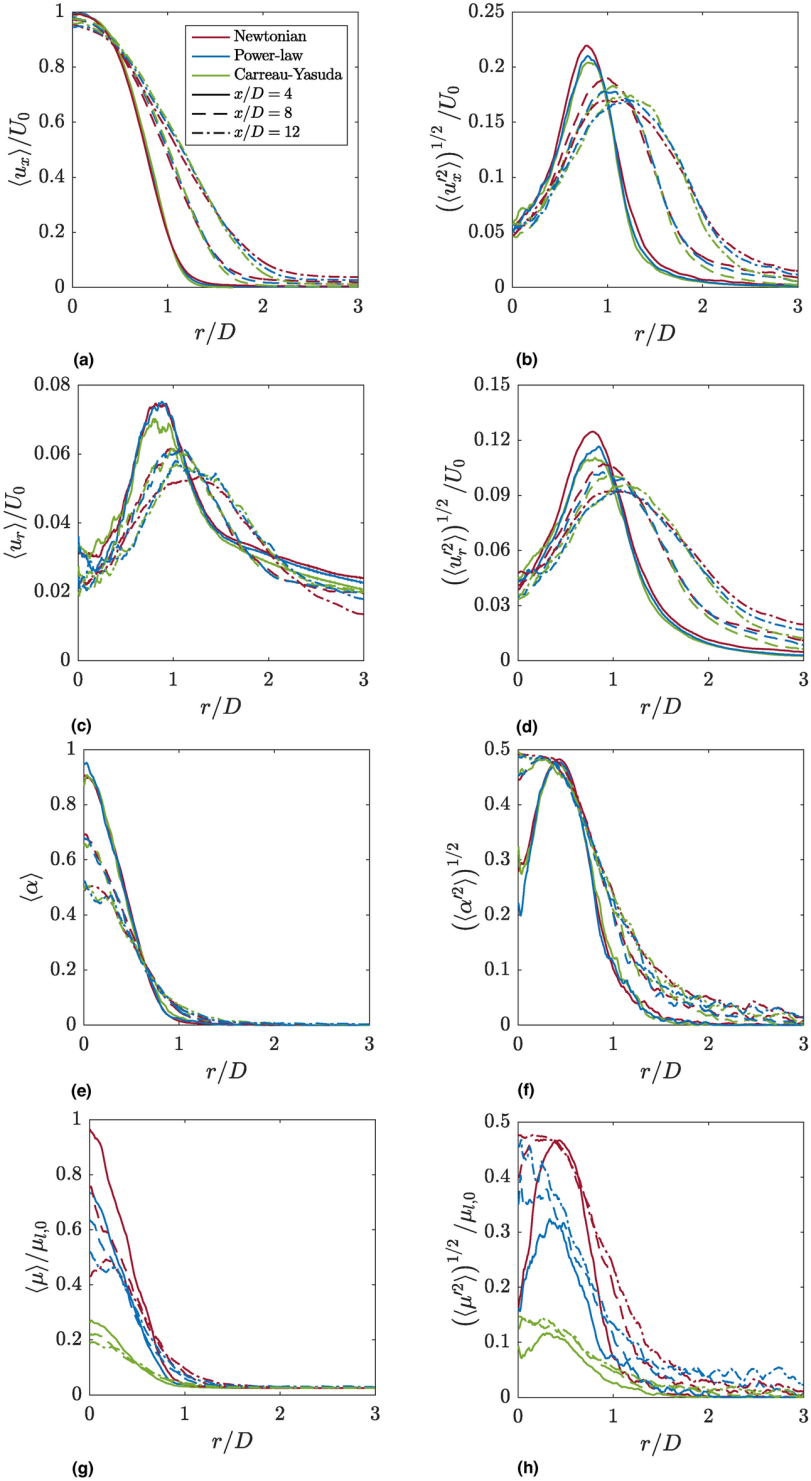
First- and second-order flow statistics at different axial positions. Depicted are (**a**) the mean axial velocity $\langle u_x\rangle$, (**b**) the root mean square (RMS) of the axial velocity fluctuations $u'_x$, (**c**) the mean radial velocity $\langle u_r \rangle$, (**d**) the RMS of the radial velocity fluctuations $u'_r$, (**e**) the mean liquid phase volume fraction $\langle \alpha \rangle$, (**f**) the RMS of the liquid phase volume fraction fluctuations $\alpha '$, (**g**) the mean dynamic viscosity $\langle \mu \rangle$ and (**h**) the RMS of the dynamic viscosity fluctuations $\mu '$.

To compare the spray behavior of the three investigated cases, first- and second-order flow statistics are calculated at different axial positions ($x/D=4,8,12$), see Fig. 5. Shown are radially averaged profiles of the mean axial velocity $\langle u_x\rangle$, the root mean square (RMS) of the axial velocity fluctuations $u'_x$, the mean radial velocity $\langle u_r\rangle$, the RMS of the radial velocity fluctuations $u'_r$, the mean liquid phase volume fraction $\langle \alpha \rangle$, the RMS of the liquid phase volume fraction fluctuations $\alpha '$, the mean dynamic viscosity $\langle \mu \rangle$ and the RMS of the dynamic viscosity fluctuations $\mu '$. The velocity and viscosity profiles are normalized by the injection velocity $U_0$ and the dynamic viscosity of the Newtonian liquid phase $\mu _{l,0}$, respectively. The results have been obtained after reaching the statistically steady-state, and the statistics have been averaged for a time period corresponding to more than six flow-through times.

The mean and RMS profiles of the axial velocity component (Fig. 5a and b) for the three cases are relatively similar. In the near-nozzle region ($x/D=4$), the mean profiles are compact and strongly influenced by the prescribed velocity profile. With increasing axial positions ($x/D=8$ and $x/D=12$), the core jet decelerates, and the mean axial velocity profile widens, thus reflecting the breakup of the jet. As the distance from the nozzle increases, the peak values of the axial velocity fluctuations decrease, while simultaneously reaching their maximum at larger radial distances from the spray axis.

Similar observations can be made for the mean and RMS profiles of the radial velocity component (Fig. 5c and d). The peaks of the profiles are located at the same radial positions as those of the axial velocity fluctuations, indicating the region of most frequent droplet and ligament formation.

At $x/D=4$, the mean liquid phase volume fraction (Fig. 5e) is close to one at the jet axis in all investigated cases, thus indicating the presence of a relatively compact spray core. Further away from the jet axis, the mean liquid phase volume fraction decreases as the jet core disintegrates into droplets and ligaments. At $x/D=8\ \text {and}\ x/D=12$, $\langle \alpha \rangle$ exhibits significantly lower values on the spray axis. This reflects the rapid breakup of the sprays investigated in the present work, which is caused by the combined action of liquid turbulence prescribed at the inflow and strong aerodynamic forces due to the low liquid-to-gas density ratio. It is evident that the shear-thinning behavior of the liquid phase barely influences the velocity and the liquid phase volume fraction statistics for the investigated setup.

The mean and RMS profiles of the dynamic viscosity (Fig. 5g and h) in the Newtonian spray are dictated by the mean and RMS profiles of the liquid phase volume fraction (Fig. 5e and f), as $\mu$ is linearly interpolated on the basis of $\alpha$. On the contrary, the profiles in the shear-thinning cases are notably influenced by the non-Newtonian character of the liquid phase. In general, both shear-thinning sprays exhibit a lower mean dynamic viscosity and weaker absolute viscosity fluctuations (stronger relative viscosity fluctuations). At $x/D=12$, however, the mean dynamic viscosity profiles for the Newtonian and the power-law jets are very similar, which is consistent with the instantaneous local Ohnesorge numbers. The weaker absolute viscosity fluctuations can partly be explained by the lower mean values of the liquid phase viscosity. These effects are more pronounced in the Carreau-Yasuda spray, as it exhibits a stronger shear-thinning character, see Fig. 1.

**Fig. 6:**
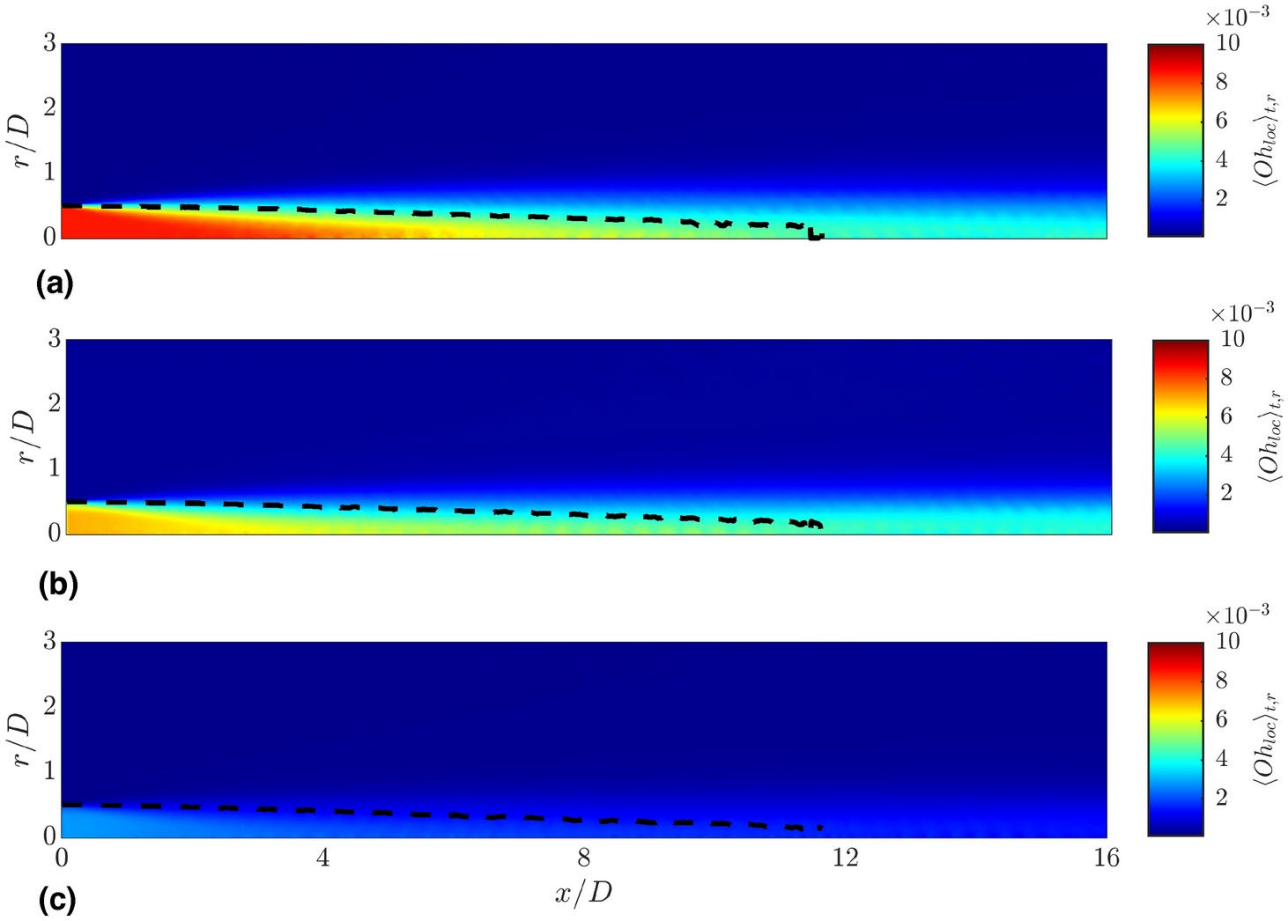
Radial and temporal average of the local Ohnesorge number. The black dashed line marks the averaged location of the gas-liquid interface defined as $\langle \alpha \rangle _{t,r}=0.5$. (**a**) Newtonian; (**b**) Power-law; (**c**) Carreau-Yasuda.

In addition to the mean dynamic viscosity profiles shown in Fig. 5g, Fig.  6 presents the radial and temporal average of the local Ohnesorge number, highlighting the variation in local dynamic viscosity in the $x-r$ plane. Again, the results have been obtained after reaching the statistically steady-state. The averaged location of the gas-liquid interface given by $\langle \alpha \rangle _{t,r}=0.5$ is marked by the black dashed line. For the Newtonian case, the local Ohnesorge number reaches its maximum in the region $0< x/D < 4$ due to the presence of a compact liquid core. In axial direction, $\langle Oh_{loc} \rangle$ decreases due to the breakup and disintegration of the jet, which results in a smearing of the $\langle \alpha \rangle$ field. The power-law case displays a lower $\langle Oh_{loc} \rangle$ in the core jet close to the nozzle, reflecting the lower dynamic viscosity compared to the Newtonian case. This is consistent with the profiles shown in Fig. 5g. However, results further downstream ($12< x/D < 16$) are comparable to the Newtonian case. A further significant decrease of $Oh_{loc}$ compared to the Newtonian and power-law cases is observed for the Carreau-Yasuda jet, which exhibits strongly reduced viscosities throughout the entire spray region.

To evaluate the volume fraction field in a more quantitative manner, the spray angles for the three cases are calculated using the mass-density based approach proposed by Balewski^41^. Starting from the jet axis, the liquid mass is integrated in radial direction at different axial positions. Once a circular cross-section contains a specified percentage $\beta$ of the mass that a cross-section of an undisturbed liquid jet would contain, the radius of this circular region is stored. After determining this radius at each examined axial position, a straight line is fitted through these values, which then determines the spray angle. Following the work of Balewski^41^, a threshold mass of $\beta =70\%$ is used. The results for the spray angles are given in Table 3. A slight increase in the spray angle is observed for stronger shear-thinning behavior of the flow. This could be due to the production of larger droplets further downstream in the shear-thinning cases, as seen in Figs. 2b and c. The spray angle characterizes the intensity of the jet breakup and thus is an important quantity for evaluating the spatial distribution of liquid mass in the downstream region. A lower spray angle increases the likelihood of droplet collision and coalescence, as the droplet collective remains more dense, while a higher spray angle results in a wider coverage area of the droplets. Consequently, the slight increase of the spray angle observed for both shear-thinning cases could marginally influence droplet collision and coalescence events. In applications such as agricultural spraying, where non-Newtonian fluids are commonly used, a slightly larger spray angle could improve droplet distribution efficiency by increasing the coverage area.

**Table 3:** Calculated spray angle for all investigated cases using a mass-density approach at a threshold mass of $\beta =70\%$.

Case	Spray angle [$^{\circ }$]
Newtonian	$3.12$
Power-law	$3.23$
Carreau-Yasuda	$3.41$

### Droplet characteristics

In this subsection, the characteristics of primary droplets newly formed from the core jet, such as droplet volumes and shapes, are discussed and compared with the characteristics of other droplets within the computational domain. All droplets with an equivalent spherical diameter of $d_d=(6V_d/\pi )^{1/3} < 2\Delta$, where $V_d$ denotes the droplet volume, have been excluded from the analysis, as interface capturing methods are unable to geometrically resolve their shape^15,42,43^.

**Fig. 7:**
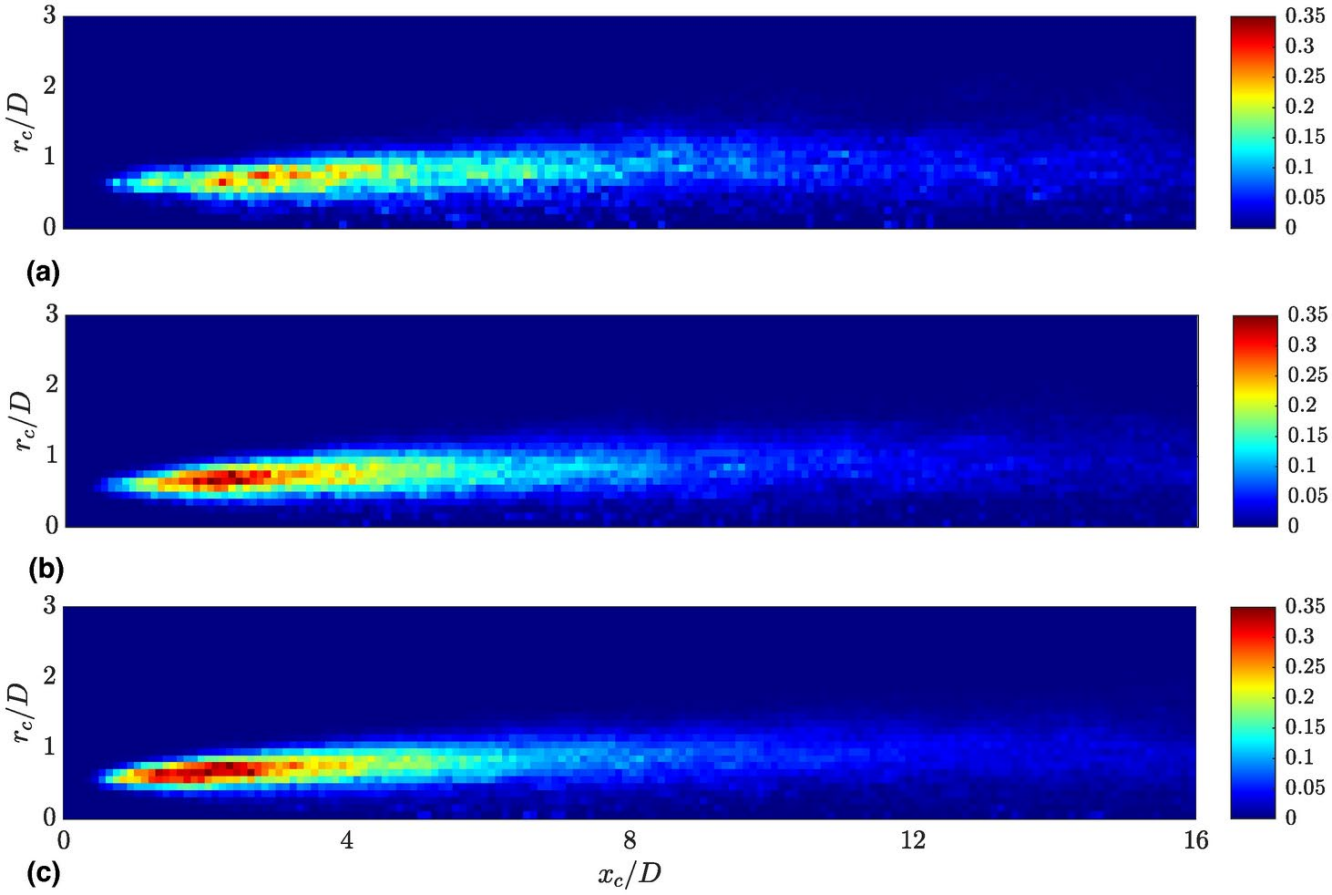
Joint probability density function (PDF) for the axial and radial positions of the detected primary droplets, representing the spatial distribution of droplet formation events. The probability density for the formation of primary droplets shifts towards the nozzle region with increasing shear-thinning behavior of the liquid phase. (**a**) Newtonian; (**b**) Power-law; (**c**) Carreau-Yasuda.

Figure 7 shows the joint probability density function (PDF) of the radial ($r_c$) and axial ($x_c$) coordinate of the center of mass for the detected primary droplets. This corresponds to the spatial distribution of droplet formation events from the core jet, which is indicated by the colormap. In direct proximity of the inlet, no formation of primary droplets is observed irrespective of the case. For the Newtonian case, the majority of the primary droplets are formed in the near nozzle region ($2< x_c/D < 4$). Towards the end of the computational domain, the frequency of primary droplet production events decreases. Compared to the Newtonian case, the probability density for the formation of primary droplets is shifted towards the nozzle region for both shear-thinning cases. This effect is particularly pronounced in the Carreau-Yasuda case. This could be caused by the high shear rates close to the nozzle region, resulting in a lower viscosity at the interface and thus an enhanced breakup. This phenomenon is known from studies varying the jet Reynolds number. For increasing Reynolds numbers, which can be achieved by decreasing the liquid phase viscosity, a more rapid formation of droplets is observed^7^.

**Fig. 8:**
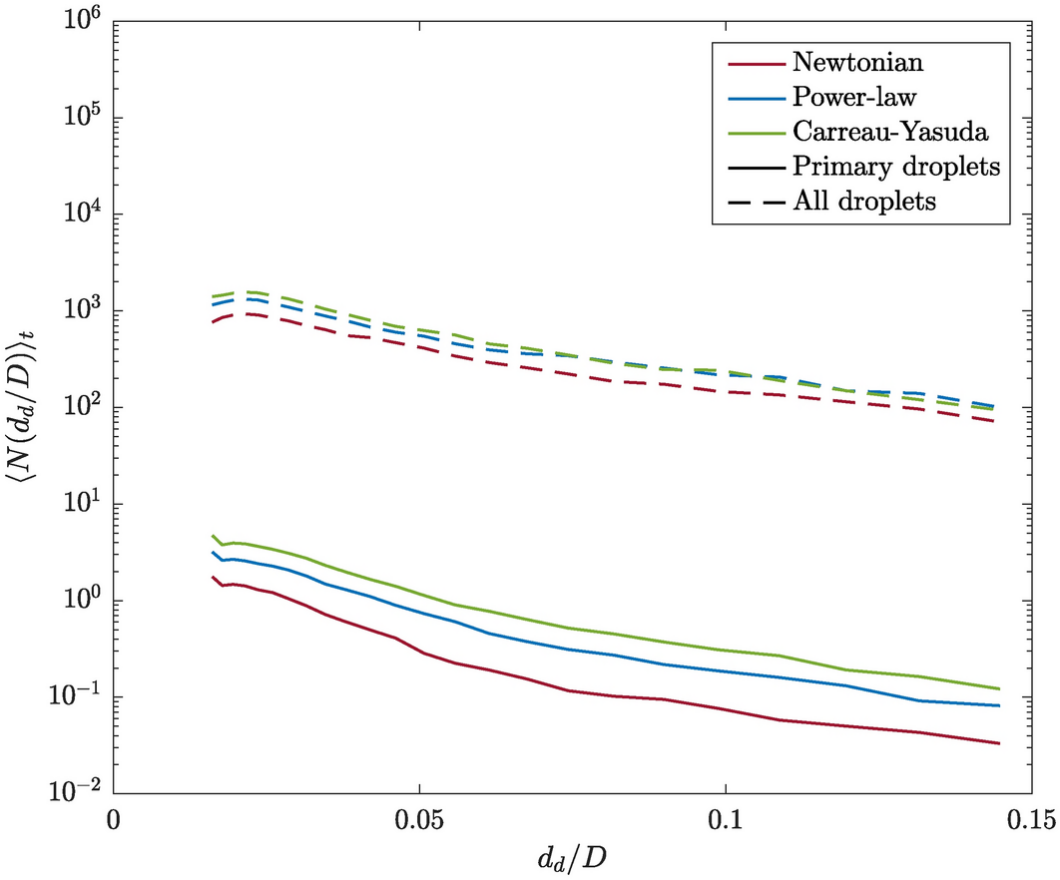
Distribution of droplet diameters of the primary droplets and all other droplets in the domain, given by the number of droplets over the droplet diameter, for all investigated cases. The comparison reveals a slight increase in droplet numbers for the shear-thinning cases.

The distribution of droplet diameters for all investigated cases is shown in Fig. 8. A comparison is made between the primary droplets and the complete droplet collective, which, by definition, includes the primary droplets. In comparison to the other cases, the Carreau-Yasuda jet produces a slightly greater number of smaller droplets. This observation applies to both the primary droplets and the entire droplet collective within the domain. The Newtonian case results in the lowest number of droplets for both categories, while the power-law spray generates more large droplets compared to the other cases. As the velocity and volume fraction statistics presented in Fig. 5 are nearly identical for all cases, it can be concluded that the shear-thinning behavior of the flow leads to a slightly increased number of droplets. These findings are consistent with a study conducted by Ling et al.^44^, which investigated the impact of fuel properties on the atomization process. In their work, the authors demonstrate that the higher viscosity of a Biodiesel jet results in a lower number of droplets compared to a Diesel jet with a lower viscosity. The droplet size distribution plays a crucial role, e.g., in agricultural sprays, which are often characterized by a shear-thinning behavior. Compared to larger droplets, smaller droplets are less effective at penetrating the canopy and tend to be more prone to wind drift. In applications such as pesticide sprays, this could lead to environmental problems. However, they provide a good coverage^45^. Thus, a compromise between minimizing wind drift while maximizing canopy penetration and coverage is essential for agricultural sprays. Similar trade-offs are also found in applications such as medical sprays or spray painting.

**Fig. 9:**
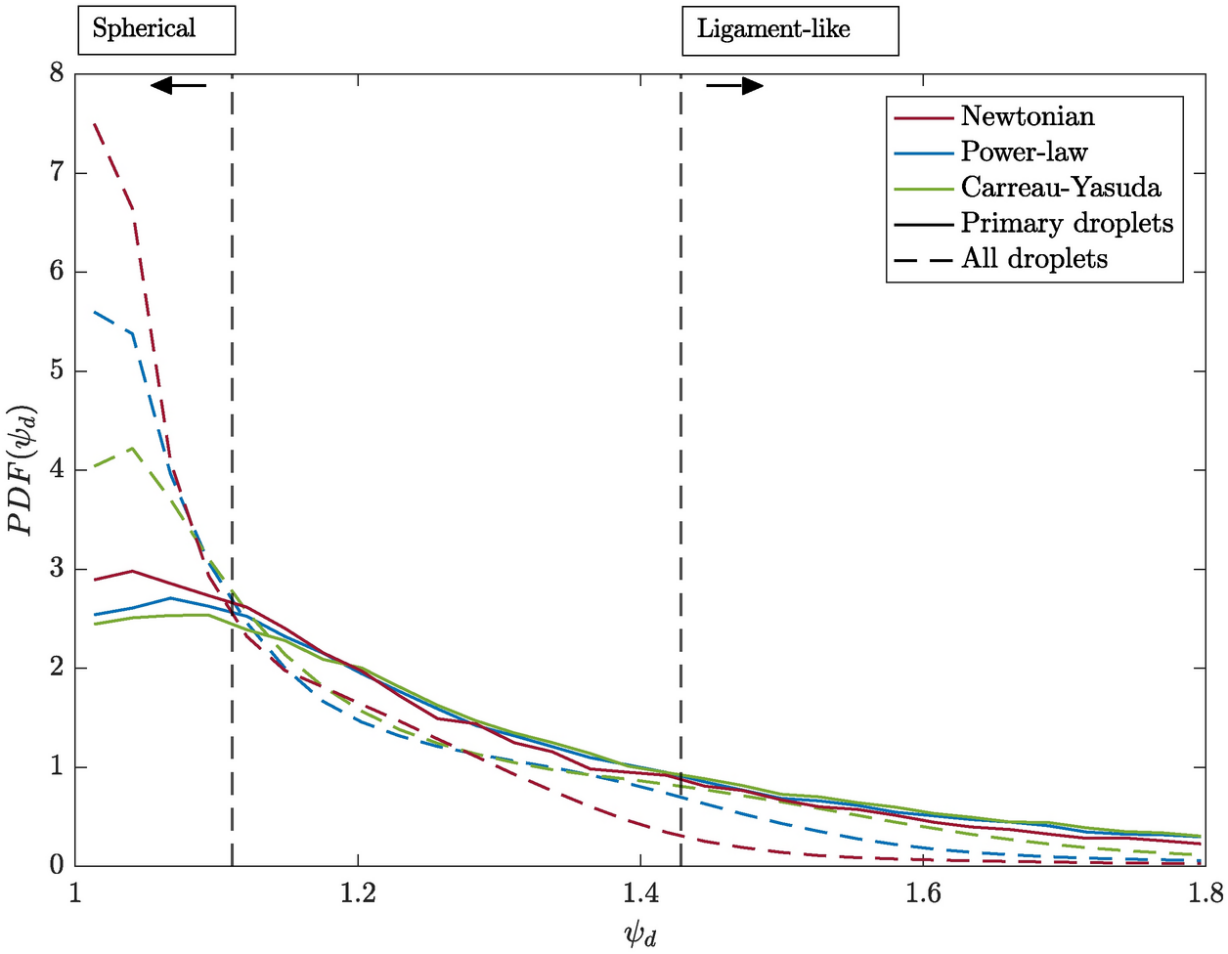
Probability density function (PDF) of the droplet deformation of the primary droplets and all other droplets in the domain for all investigated cases.

In the next step, the droplet shapes are investigated. For this purpose, the droplet deformation $\psi _d$ is defined as the ratio of the droplet surface area $A_d$ and the surface area of a sphere with an identical droplet volume $A_{d,sphere},$ thus leading to^9^

12 $$\begin{aligned} \psi _d=\dfrac{A_d}{A_{d,sphere}}=\dfrac{A_d}{\pi ^{1/3}(6V_d)^{2/3}}. \end{aligned}$$

Following the definition of Pairetti et al.^43^, droplets with $\psi _d < 1.11$ are assumed to have a quasi-spherical shape, whereas droplets with $\psi _d > 1.43$ are assumed to be ligament-like. Figure 9 illustrates the PDF for the droplet deformations of all investigated cases. Once again, a comparison is made between the detected primary droplets and the complete droplet collective present in the computational domain. In all three cases, the most frequent droplet shape detected throughout the domain is quasi-spherical. A notable amount of droplets exhibits deformations, but only a small minority is ligament-like. While the most frequently observed droplet shape for primary droplets is also spherical, they generally show stronger deformations than the entire droplet collective for all three cases. These differences in the PDFs between the primary droplets and the entire droplet collective within one case can be explained by evaluating the droplet’s Capillary number $Ca_d=(\mu _g \mid {\textbf {u}}^{rel}_{d} \mid )/\sigma.$ The Capillary number describes the ratio of viscous drag to surface tension forces, and $Ca_d<<1$^9^ indicates high surface tension forces, which act on primary droplets and thus makes them more spherical. In addition, it is observed that, compared to the shear-thinning jets, the Newtonian jet produces the most spherical primary droplets. With increasing importance of the shear-thinning flow behavior, and thus lower interfacial viscosity values, the primary droplets exhibit stronger deformations and even ligament-like shapes. This can potentially be explained by the droplet Ohnesorge number $Oh_d=\mu _l/(\sqrt{\rho _l \sigma D_d})$, which relates the viscous forces to inertial and surface tension forces. As droplet formation is assumed to be dominated by stretching and rupture of ligaments^5,12,15^, the primary droplets produced tend to initially exhibit a stretched shape. If a newly formed droplet is initially deformed, the surface tension acts on the drop to pull it back towards a spherical form. This surface tension force is converted into a momentum force, which could cause additional deformation and oscillation of the drop. The higher the viscosity of the drop, the greater its Ohnesorge number, resulting in more damped oscillations. This is because the viscous forces counteract the oscillations^46^. This finding is consistent with recent findings of Li et al.^47^, who investigated the secondary breakup of shear-thinning droplets in different breakup regimes. The authors observed enhanced droplet deformation for decreasing power-law indexes *n* and thus decreasing liquid phase viscosities. Moreover, this is also consistent with the general idea behind the Taylor Analogy Breakup (TAB) model^48^, which suggests an analogy between an oscillating and distorting droplet and a spring-mass system. In this context, the damping force is represented by liquid viscosity effects.

## Summary and conclusions

In this DNS study, we investigate the effect of shear-thinning behavior of the liquid phase on the primary atomization of non-Newtonian jets. The focus is on the characteristics of primary droplets newly formed from the core jet, such as droplet sizes and shapes. For the identification of primary droplets at the instant of their formation a very recently suggested algorithm^9^ is used and applied for the first time to non-Newtonian sprays. Overall, three cases are investigated, with a Newtonian case serving as the reference. Two shear-thinning cases are investigated using two different models for the viscosity, namely the power-law and the Carreau-Yasuda models. The Carreau-Yasuda spray exhibits a stronger shear-thinning behavior than the power-law case. Due to a lack of detailed experimental data, the two shear-thinning fluids investigated in this study are hypothetical fluids that serve as simplified models and do not represent a specific real-world application. Future work will extend the current analysis to specific fluids and real technical application scenarios.

To the authors’ best knowledge, this work is the first to present mean flow statistics for non-Newtonian atomization. While the local dynamic viscosity of the shear-thinning jets is strongly influenced by the local shear rate, the first- and second-order statistics of the axial and radial velocity, as well as the liquid phase volume fraction statistics (Fig. 5a-f), are very similar to those of the Newtonian case.

Unlike the presented flow statistics, which have been radially and temporally averaged and therefore do not provide insight into the instantaneous flow behavior, the analysis of the droplet characteristics reveals that the instantaneous and local behavior of the viscosity at the interface plays a certain role. As indicated by the spatial distribution of the detected primary droplets (Fig. 7), the majority of primary droplets are formed relatively close to the nozzle in all cases. However, it has been observed that for increasing influence of the shear-thinning behavior, and thus decreasing values of the liquid phase viscosity, the region of maximum droplet formation is shifted towards the nozzle. Although the differences are not pronounced, the highest number of small droplets is observed for the Carreau-Yasuda spray, while the Newtonian case results in the lowest total droplet count (Fig. 8). The largest droplets are produced by the power-law spray. Additionally, it was observed that a decrease in viscosity at the interface, as seen in the instantaneous local Ohnesorge plots, leads to a slight increase in the detected number of droplets, which is consistent with the results of earlier studies investigating Newtonian jets with different liquid phase viscosities^44^. The majority of droplets exhibit relatively spherical shapes, which is attributed to strong surface tension forces (Fig. 9). However, lower viscosities at the interface, which are observed for the shear-thinning cases, are found to lead to less spherical and more ligament-like droplets. The present analysis reveals that the droplet mass is relatively small compared to the mass of the liquid core. Thus, while there are differences in the droplets statistics, such as their shapes and spatial distribution, the droplets produced barely impact momentum transfer and exchange. This is reflected by the quasi unchanged first- and second-order velocity and volume fraction statistics.

The current analysis provides a foundation for future physics-driven spray modeling, particularly for shear-thinning sprays. A comparison between the obtained numerical results and experimental data is not within the scope of the current study and thus is a promising topic for future work. It should be noted, however, that experimental identification of droplets at the instant of their formation, as well as the investigation of their characteristics, remains challenging due to the limited optical accessibility in the dense near-nozzle region^49^. As a next step, the analysis will be extended to thixotropic fluids, where the viscosity is is not only a function of the shear rate, but additionally a function of the time.

## Data Availability

Correspondence and requests for materials should be addressed to M.A.
